# Hypoxia within the glioblastoma tumor microenvironment: a master saboteur of novel treatments

**DOI:** 10.3389/fimmu.2024.1384249

**Published:** 2024-06-26

**Authors:** Lisa Feldman

**Affiliations:** Division of Neurosurgery, City of Hope National Medical Center, Duarte, CA, United States

**Keywords:** glioblastoma, hypoxia, tumor microenvironment, novel treatments, immunotherapy

## Abstract

Glioblastoma (GBM) tumors are the most aggressive primary brain tumors in adults that, despite maximum treatment, carry a dismal prognosis. GBM tumors exhibit tissue hypoxia, which promotes tumor aggressiveness and maintenance of glioma stem cells and creates an overall immunosuppressive landscape. This article reviews how hypoxic conditions overlap with inflammatory responses, favoring the proliferation of immunosuppressive cells and inhibiting cytotoxic T cell development. Immunotherapies, including vaccines, immune checkpoint inhibitors, and CAR-T cell therapy, represent promising avenues for GBM treatment. However, challenges such as tumor heterogeneity, immunosuppressive TME, and BBB restrictiveness hinder their effectiveness. Strategies to address these challenges, including combination therapies and targeting hypoxia, are actively being explored to improve outcomes for GBM patients. Targeting hypoxia in combination with immunotherapy represents a potential strategy to enhance treatment efficacy.

## Introduction

Glioblastoma (GBM) is an aggressive and devastating primary brain tumor characterized by a high proliferation rate, infiltrative growth, and resistance to traditional interventions. It is the most common primary brain tumor in adults, with an incidence ranging from two to five cases per 100,000 people (1). GBM accounts for approximately 48.3% of all primary malignant brain tumors (2) and affects individuals across various age groups. Its prevalence increases with age, peaking in the 75–84 years of age group, with a median age of 64 at diagnosis (3). GBM is more common in men than women, and more prevalent in Caucasians compared to Afro-Americans, Africans, American Indians, and Asians (3). This malignancy primarily affects the cerebral hemispheres, particularly in subcortical white matter and deep grey matter of the frontal and temporal lobes, although it can arise in any part of the brain (4).

GBM tumors are a type of diffuse astrocytic tumor classified as grade IV by the World Health Organization (WHO). According to the 2021 WHO classification of CNS tumors, they are now required to express isocitrate dehydrogenase (IDH-wild type) to be classified as GBM (5). GBM tumors are generally divided into primary and secondary tumors with the former arising from either progenitor or neural stem cells (90% of GBM), and the latter transforming from pre-existing, less aggressive tumors (10%) (6). Regardless, the pathogenesis of GBM involves wildly variable and complex genetic and molecular alterations. Amplification of the epidermal growth factor receptor (EGFR) gene, mutations in the IDH1 and IDH2 genes, and loss of tumor suppressor genes such as TP53 and PTEN serve as the most identified genetic abnormalities in GBM (7).

These molecular aberrations contribute to uncontrolled cell growth, angiogenesis, and invasion into the surrounding brain tissue (8). Therefore, current treatment modalities for GBM typically involve a combination of surgery, radiation therapy, and chemotherapy. The goal of surgery is to remove as much tumor as possible without compromising neurological function. Adjuvant radiation therapy follows surgical debulking by delivering targeted beams to the remaining cancer cells in the tumor bed to delay recurrence. Concurrently, chemotherapy, usually temozolomide (TMZ), is administered during and after radiation to enhance the treatment outcomes. Despite these aggressive treatment approaches, GBM inevitably recurs, underscoring the challenge of eradicating residual tumor cells and the need for innovative therapeutic interventions. Because these standard therapies only extend survival an average of 15 months (9), with less than 30% of patients living 2 years (10), neuro-oncologists are eager to develop new therapies.

Here, we review major challenges to developing successful treatments against GBM and focus on how hypoxia contributes to these obstacles.

## The heterogeneity of GBM

Designing novel therapies against GBM has proven to be remarkably challenging due to the extensive variability of the GBM tumor microenvironment (TME) within brain tissue. GBM tumors metastasize outside the central nervous system (CNS) in less than 0.2% of cases (11), suggesting they require the unique milieu of brain parenchyma to survive. Together, the variety of unique resident brain cells, the favorable blood supply with high delivery of oxygen and nutrients to the brain, and the blood-brain barrier that creates a relatively immune-privileged state create an environment in which GBM tumor cells thrive. Once GBM cells develop and migrate into adjacent tissues, these tumors develop prototypical heterogeneity both within single tumors as well as across tumors in multifocal disease.

GBM is characterized by an overwhelming level of intratumoral heterogeneity, manifesting at various biological levels. At the genetic level, extensive genomic analyses have revealed diverse molecular alterations within individual tumors and across different lesions of multifocal GBM cases. The Cancer Genome Atlas (TCGA) research network has played a pivotal role in uncovering the genetic landscape of GBM, identifying mutations in genes such as EGFR, TP53, and PTEN that contribute to tumor heterogeneity (12, 13). This genetic diversity not only influences the aggressive behavior of GBM, but also poses challenges for developing targeted therapies.

In addition to genetic heterogeneity, GBM tumors exhibit profound cellular diversity. Amongst a background of mutated glial cells demonstrating rapid proliferation, migration, and infiltration throughout normal brain tissue, the GBM TME also includes cancer stem cells (CSCs) that demonstrate multipotent differentiation and true self-renewal properties (14, 15). Stromal cells, vascular endothelial cells, and both residing and infiltrating immune cells further contribute to specific niches within the TME (16). Traditionally, intratumoral heterogeneity is considered a result of distinct genetic alterations within various tumor cells (17). However, it is more likely the complex interaction of each of these cell types within the extracellular matrix (ECM) that contributes to heterogeneity. For example, within these specific niches, the proliferating or CSC-like tumor cells dynamically interact with resident parenchymal cells, such as microglia and macrophages, to orchestrate a unique milieu. The microscopic result of these various TMEs invariably leads to necrotic regions, endothelial proliferation around vessels, and hypoxic areas (16), all of which are classical pathological findings of GBMs.

The spatial heterogeneity observed within GBM tumors further adds to the complexity of the disease. Intra-tumoral variations in oxygen levels, nutrient availability, and blood flow create distinct microenvironments within the tumor mass. These variations lead to the formation of hypoxic regions, which are known to influence tumor progression, treatment resistance, and the emergence of more aggressive phenotypes (18).

To summarize, the heterogeneity of GBM is a complex phenomenon that covers various aspects like genetics, cells, microenvironment, and spatial diversity. The challenges posed by this heterogeneity highlight the intricate nature of GBM biology, which emphasizes the requirement for personalized and targeted therapeutic approach. The advancements in genomic profiling, single-cell analysis, and a deeper understanding of the TME pave the path for developing innovative strategies to address the diverse components that contribute to GBM heterogeneity.

## The immunosuppressive GBM TME

**Figure 1:**
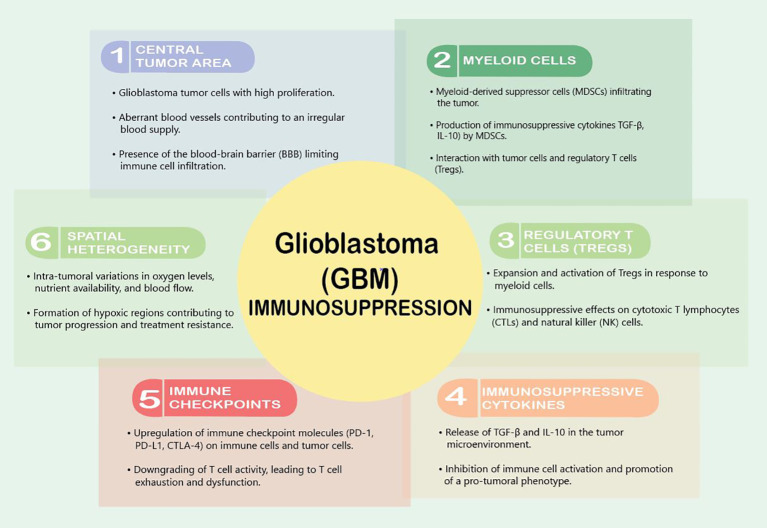
Factors contributing to immunosuppression in GBM.

GBM tumors are notorious for creating an immunosuppressive microenvironment that facilitates their growth and evades the host’s immune response. This immunosuppressive nature is orchestrated by a complex interplay of various cellular and molecular components within the TME (**Figure 1**). One prominent feature is the recruitment and activation of immunosuppressive cells, including regulatory T cells (Tregs) and myeloid-derived suppressor cells (MDSCs), which inhibit the activity of cytotoxic T lymphocytes (CTLs) and natural killer (NK) cells, crucial effectors of anti-tumor immunity (19, 20). The abundance of these immunosuppressive cells contributes to the establishment of an immune-tolerant milieu that shields GBM from immune-mediated destruction.

VEGF is the master regulator of this immunosuppression. It downregulates intercellular adhesion molecule 1 (ICAM1) and vascular cell adhesion molecule 1 (VCAM1), adhesion molecules that reduce the infiltration of T-effector cells, activate antigen-specific regulator T cells (Tregs), and inhibit the maturation of dendritic cells (DCs) (21). Interestingly, a variety of brain tumors induce reduced expression of cell membrane sphingosine-1-phosphate receptor (S1P1), which causes trapping of T cells in bone marrow, and therefore actively induces an immunosuppressive state (22). Furthermore, Andaloussi et al. found a dramatic reduction in CD4+ and CD8+ cells, a reduction in the size of the thymus, and a decrease in thymic cellularity in the brains of mice implanted with murine glioma GL261 cells (23).

Upon reaching the GBM TME, T cells encounter a harshly immunosuppressive local state due to high concentrations of immunosuppressive modulators such as transforming growth factor β (TGF-β), prostaglandin E-2 (PGE2), and interleukin 10 (IL-10). This cytokine milieu shifts microglia and tumor-infiltrating myeloid cells to a quiescent phenotype that dampens immune responses and supports tumor growth (24). Additionally, GBM tumors exploit the expression of immune checkpoint molecules to dampen the anti-tumor immune response. Programmed cell death protein 1 (PD-1) and its ligand PD-L1, along with cytotoxic T-lymphocyte-associated protein 4 (CTLA-4), serve as key immune checkpoints that, when engaged, downregulate T cell activity (25, 26). GBM cells and immune cells within the TME upregulate these checkpoint molecules, leading to T cell exhaustion and dysfunction (26). The upregulation of these immune checkpoints contributes to the immune escape mechanisms employed by GBM tumors, limiting the effectiveness of immunotherapeutic interventions.

Myeloid cells play a pivotal role in establishing an immunosuppressive microenvironment within GBM tumors. myeloid cells, including M2 macrophages, immature monocytes, and myeloid-derived suppressor cells (MDSCs), are the most common non-malignant cells identified in the GBM TME (27). Among these cells, MDSCs are frequently recruited to the GBM microenvironment, where they contribute to the inhibition of both innate and adaptive immune responses, dampening the activity of cytotoxic T lymphocytes (CTLs) and natural killer (NK) cells, which are crucial effectors of anti-tumor immunity (20). These cells play a major role in tumor-mediated immunosuppression through further immune checkpoint molecule expression and anti-inflammatory cytokine release (28). Upregulation of programmed death-ligand 1 (PD-1) on tumor-infiltrating myeloid cells is a crucial GBM-derived method of immune evasion (29, 30). GBM cells themselves release interleukin 6 (IL-6), which is necessary for myeloid PD-L1 induction through a signal transducer and activator of transcription 3 (STAT3)-dependent fashion and disrupting IL6 signaling enhances immune-mediated antitumor effects against GBM (28).

L. Pang et al. recently identified Kunitz-type protease inhibitor TFPI2 as a crucial player in connecting various GBM cell populations, including self-renewing stem cells and immunosuppressive microglia. TFPI2 enhances stem cell renewal and tumor growth by activating the c-Jun N-terminal kinase–signal transducer and activator of transcription (STAT)3 pathway. Simultaneously, secreted TFPI2 triggers the polarization of immunosuppressive microglia via STAT6 signaling. In human GBM, TFPI2 is associated with stemness, immunosuppression, increased microglia, and poor prognosis. Inhibiting the TFPI2-CD51-STAT6 pathway both activates T cells and improves the efficacy of anti-PD1 immunotherapy against GBM in mice (31). Moreover, this same group also characterized legumain (LGMN) as a key protease that is transcriptionally regulated by HIF1α and enriched in tumor-associated macrophages. Increased LGMN activates the GSK-3β-STAT3 signaling pathway to promote TAM immunosuppression. Inhibiting HIF1α and LGMN enhances CD8+ T cell- anti-tumor immunity, impairs GBM tumor progression, and improves immunotherapy responses (anti-PD1 therapy) in mice (32). Altogether, these data suggest that targeting TFPI2, as well as HIF1α and LGMN, are exciting strategies to combat GBM.

Furthermore, myeloid cells within the GBM microenvironment actively promote the expansion and activation of regulatory T cells (Tregs). Tregs are a subset of T lymphocytes that play a crucial role in immune tolerance and homeostasis. The interaction between myeloid cells and Tregs creates a self-reinforcing loop of immunosuppression as Tregs, in turn, support the survival and function of MDSCs. This collaboration contributes to establishing an immunosuppressive network that shields GBM from effective immune surveillance and clearance (19).

Strategies to counteract the immunosuppressive effects of myeloid cells in GBM are actively being explored in the field of cancer immunotherapy. Targeting MDSCs or modulating their function to reduce their immunosuppressive capacity represents a potential avenue for enhancing the anti-tumor immune response. However, the remarkable plasticity and adaptability of myeloid cells in response to the GBM microenvironment pose significant challenges to the development of effective therapeutic interventions. A comprehensive understanding of the intricate interactions between myeloid cells and the immune system within the GBM TME is crucial for devising innovative and targeted immunotherapeutic strategies.

The blood–brain barrier (BBB) plays an active role in generating immunosuppression within the GBM TME. The BBB is a specialized endothelial barrier that separates the bloodstream from the brain tissue, tightly regulating the passage of toxins, drugs, and immune cells into the CNS. Altogether, there are at least five ways in which the BBB affects the neuroimmune status: through BBB permeability, modulation of the BBB transporters, removal of immunoreactive substances by cells of the BBB, transfer of immune cells through the BBB into the brain, and secretion of immunoreactive substances by cells of the BBB (33). In GBM, the stability of the BBB reflects the degree to which the BBB contributes to the establishment of an immunosuppressive milieu within the tumor. The selective permeability of the BBB limits the entry of circulating immune cells, including cytotoxic T lymphocytes (CTLs) and natural killer (NK) cells, into the tumor site. This restriction hampers the effectiveness of the anti-tumor immune response, as these cells are crucial effectors in recognizing and eliminating cancer cells (34). This restrictive behavior also prevents the delivery of many therapeutics into the tumor. Therefore, considerable efforts have been made to modulate or bypass the BBB, either invasively or non-invasively, for the purpose of drug delivery (35). However, the delicate balance between protecting the brain from potential immune-mediated damage and promoting an effective anti-tumor immune response poses a significant challenge in developing targeted and safe therapeutic approaches.

The BBB traditionally was thought to contribute to a relatively immune-privileged state of the brain, creating an environment where immune surveillance is inherently restricted. This immune-privileged status of the brain was thought to be related to the lack of a traditional lymphatic drainage system, limiting immune cells to efficiently traffic to and from the CNS and target tumor cells (36). More recently, however, the brain is no longer considered a truly immune-privileged organ, as microglia act as resident antigen-presenting cells and allow T cells to traffic in and out of the CNS (24). In GBM, this immune privilege is exploited by the tumor, enabling it to thrive in an environment that is less susceptible to immune-mediated destruction (36). The leaky BBB in GBM also leads to the influx of pro-tumoral immune cells into the TME, such as infiltrating MDSCs and T_regs_. Once infiltrated in the tumor, these cells contribute to the immunosuppressive network by suppressing effector immune responses and promoting a tumor-favorable microenvironment (20, 37).

The BBB also regulates the transport of immune-modulating molecules into the brain. Endothelial cells within the BBB express various transporter systems on their surface, encompassing both active transport mechanisms and those functioning through facilitated diffusion. The roles of certain transporters undergo alterations in the context of neuroinflammation and are subject to modulation by signaling molecules, with the P-glycoprotein (Pgp) protein being a notable example. Pgp is a BBB efflux transporter that limits drug entry into the CNS through interactions with diverse ligands, including protease inhibitors, opiates, antiepileptics, cyclosporins, glucocorticoids, aldosterone, dexamethasone, and calcium channel blockers. Indeed, Pgp acts as a gatekeeper of the CNS as its functionality undergoes changes during inflammation, primarily resulting in the suppression of its transport activity *in vivo*. Consequently, induction of Pgp by proinflammatory cytokines such as TNFα, IL-1β, IL-6, IL-2, and IFNγ leads to a reduction in Pgp mRNA expression in CNS endothelial cells, affecting both synthesis and activity of the transporter (38, 39).

Understanding the intricate relationship between the BBB and immunosuppression in GBM is crucial for the development of therapeutic strategies. Strategies that aim to selectively breach the BBB or modulate its permeability are actively being explored to enhance immune cell infiltration into the tumor and improve the efficacy of immunotherapeutic interventions.

## Aberrant neo-angiogenesis in GBMs

GBMs are notoriously vascular tumors in which endothelial cells support tumor growth by delivering nutrients to the tumor; however, blood vessels within GBM tumors are faulty because of their disorganized formation. GBM tumor cells, inflammatory, and stromal cells all contribute to an aggressive production of a disarrayed tangle of leaky, abnormal new blood vessels (40). Angiogenesis plays a major role in the development of these abnormal vessels; however, neovascularization via proliferation of existing endothelial cells and via hypoxia-inducible factor 1α (HIF-1α) recruitment of bone marrow-derived vascular cells also contributes to tumor angiogenesis (41). Glioma stem cells themselves also generate novel vasculature by transforming themselves into pericytes or endothelial cells (42, 43). This pro-angiogenesis drive is supported by the tumor’s inherent metabolic, inflammatory, and hypoxic conditions. Importantly, tumor cells that are often found in the periphery of the hypoxic necrotic core release unregulated levels of VEGF as well as additional paracrine factors that further lead to vascular bed inflammation and increased vascular permeability (44).

VEGF is a master regulator of the vascular aberrations in GBM, which in addition to driving faulty neo-angiogenesis, also result in the breakdown of the BBB. The BBB is comprised of astrocytes, endothelial cells, and tight junctions by pericyte feet, which together tightly regulate the transfer of molecules between the blood and brain parenchyma (45). The breakdown of the BBB results in increased vessel permeability with the influx of fluid into tumor tissue and surrounding brain tissue, resulting in cerebral edema and increased intracranial pressure. Simultaneously, the slowing of blood flow through these compromised blood vessels results in patchy oxygen delivery within the tumor bed. These focal areas of hypoxia develop from occluded vessels into pseudopallisading necrosis, a pathognomonic characteristic of GBM. These necrotic niches also recruit macrophages and other innate immune cells that further propagate angiogenesis and immunosuppression.

More recently, Pang et al., have shown that the circadian regulation of glioma stem cells alters the immunosuppressive TME in GBM tumors through paracrine and autocrine mechanisms (46, 47). One way the stem cells interact with the TME is via circadian regulation through CLOCKs, which include both transcriptional activators (such as CLOCK-BMAL1 complex) as well as inhibitors (such as cryptochrome 1 and 2, period 1,2,3 and REV-ERBα (48). CLOCK-BMAL1 complex in glioma stem cells leads to the upregulation of periostin (POSTN) that ultimately activates the TANK-binding kinase 1 (TBK1) in endothelial cells. The inhibition of POSTN and TBK1 in GBM mouse models, as well as pathological analysis in GBM patient samples, reveal that this pathway may be an interesting therapeutic strategy for targeting GBM angiogenesis (49).

## Hypoxia within GBM

GBM tumors are characterized by areas of tissue hypoxia (50–53), which not only render them more aggressive (51) but are also critical for the maintenance of glioma stem cells, the tumoral cell population that is responsible for resistance to therapies (54, 55) and tumor recurrence (56). Hypoxic and inflammatory responses overlap powerfully in the GBM microenvironment (51), as hypoxic conditions restrict cytotoxic T lymphocyte development while promoting the proliferation and expression of inflammatory cytokines (57). Although not yet completely understood, glioma stem cells, which thrive in hypoxic conditions, preferentially inhibit the proliferation of activated T cells, as compared to differentiated tumor cells (58). Hypoxic conditions within GBM tumors activate the STAT3 signaling pathway, which mediates HIF-1α and leads to immunosuppression (59). Finally, the hypoxic TME recruits immunosuppressive cells such as tumor-associated macrophages, myeloid-derived suppressor cells, and T_regs_ (60), which all reduce the immune response through the expression of inhibitory molecules on the surface of effector immune cells (51).

## New frontiers in treatment: immunotherapies

**Figure 2:**
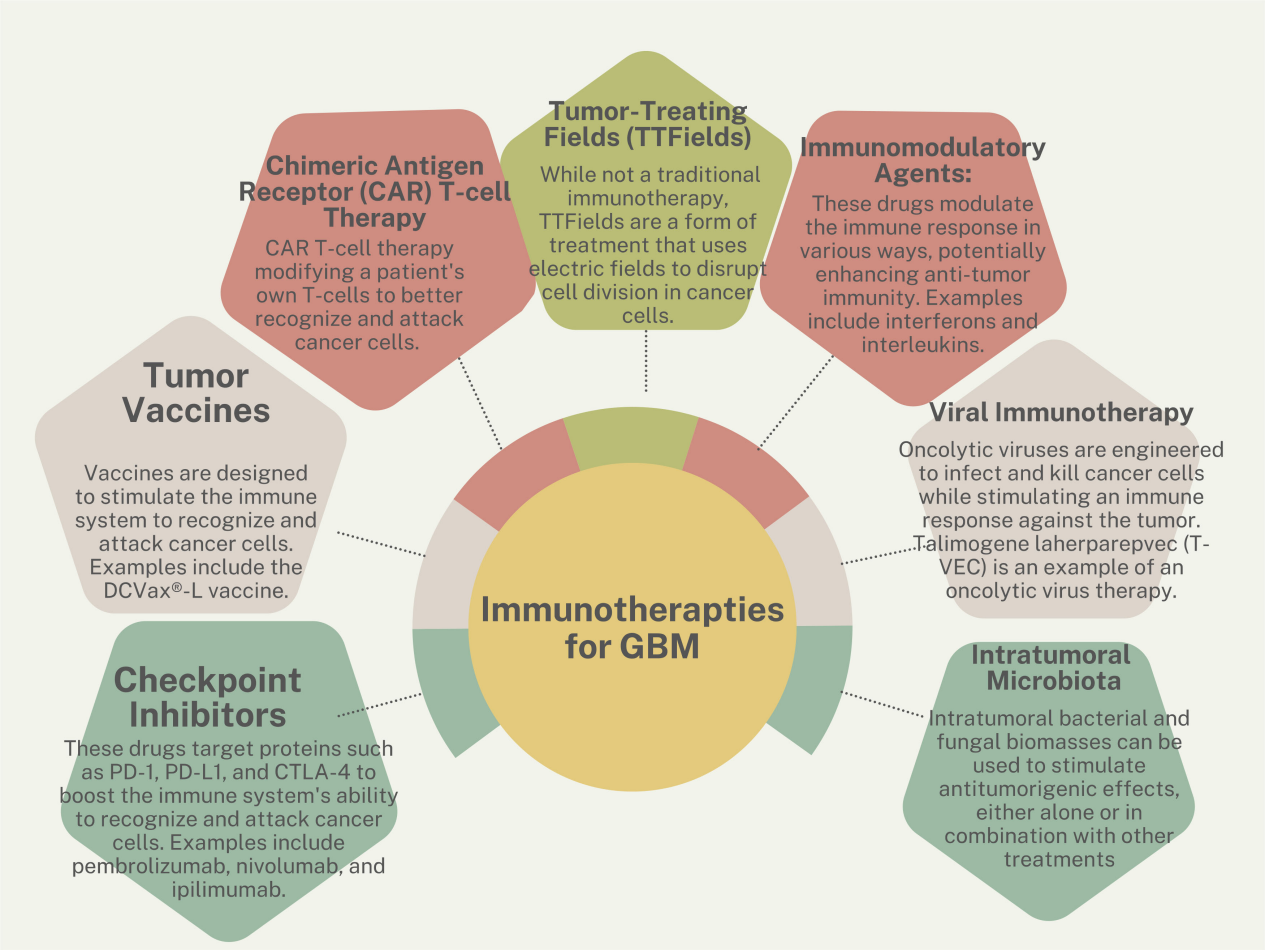
Immunotherapy strategies for GBM.

Immunotherapies are one of the newest frontiers in combating cancer (**Figure 2**). These therapies take advantage of a patient’s existing functional immune system by harvesting immune cells and retraining them to attack and kill tumor cells. These treatments have been FDA-approved for multiple blood-borne cancers (61) and are used investigationally for brain tumors. Immunotherapeutic strategies cover a wide range of treatments and include vaccines (62), adoptive cell therapy (63), monoclonal antibodies (64), oncolytic viruses (65, 66), and immune checkpoint blockers (67, 68).

### Vaccines

The field of GBM vaccine development has evolved with various approaches aimed at eliciting immune responses against tumor-specific antigens. Over two decades ago, one of the first antitumor vaccines was generated utilizing autologous dendritic cells (DCs) transduced with a replication-defective adenovirus encoding the full-length melanoma antigen MART-1/Melan-A (MART-1) to target melanoma (69). Since then, significant advancements in vaccine technology have been made to include class I-restricted peptide cocktails, multi-peptide vaccines, long peptide vaccines, peptide mimics, personalized peptide vaccines, and dendritic cell vaccines loaded with peptides, RNA, or tumor lysate.

Current clinical trials for glioma involve diverse vaccine formulations, targeting antigens like EGFRvIII, survivin, human cytomegalovirus proteins, and Wilm’s tumor protein-1 (70). Despite promising results in earlier phases, there have been challenges in translating efficacy from Phase II trials, exemplified by the EGFRvIII vaccine Rintega, which after completing the Phase III trial (ACT IV), showed that Rintega-treated patients had median overall survival of 20.4 months compared to 21.1 months in the control arm (71). Tumor progression was linked to reduced expression of EGFRvIII, indicating that Rintega triggered a distinct and potent immune response, leading to the effective elimination of GBM cells that expressed the targeted antigen. Subsequently, it was conjectured that tumors eluded immunologic regulation post-vaccination by discarding the targeted EGFRvIII antigen (72).

Heat-shock protein vaccines, particularly HSPPC-96, a dendritic cell vaccine derived from autologous tumor-derived HSP–peptide complex, have demonstrated positive immune responses in recurrent GBM patients. In a single-arm Phase II trial, HSPPC-96 was administered to patients after undergoing gross total resection of recurrent GBM. Treated patients enjoyed a median overall survival of 42.6 weeks (73). Additionally, ongoing trials explore the potential of personalized neoantigen vaccines and dendritic cell-based approaches in newly diagnosed GBM patients. The complex interplay between vaccine strategies, patient characteristics, and standard therapies necessitates careful evaluation in the quest for effective GBM immunotherapy.

### Immune checkpoint inhibitors

Brain metastases occur in 8–10% of cancer patients (74, 75), with melanoma and lung cancer having the highest cumulative incidence (76). Like patients with GBM, the prognosis for patients with brain metastases is also poor, despite multidisciplinary treatments involving surgery, irradiation, and systemic therapies. Within the last decades, there has been excellent progress in understanding how solid cancer cells evade the immune system through immune checkpoints, including CTLA-4, PD-1, and PD-L1. Blockage of these immune checkpoints with antibodies such as ipilimumab (anti-CTLA-4), nivolumab (anti-PD-1), and pembrolizumab (also anti-PD-1) have shown efficacy in various solid tumors, particularly melanoma and non-small cell lung cancer, leading to prolonged survival in extracranial disease.

To systematically evaluate the use of checkpoint inhibitors in Neuro-Oncology, a review of relevant literature was conducted following PRISMA guidelines. The search identified 88 eligible publications, including studies on GBM and brain metastases. For brain metastases, 40 studies assessed ICIs without radiotherapy, and 40 studies explored the combination of ICIs with radiotherapy. The median intracranial progression-free survival for patients with brain metastases treated with ICIs was 2.7 months, and the overall progression-free survival was 3.0 months. The median survival for these patients was 8.0 months. The studies revealed varying efficacy across different tumor types and ICI agents. For example, ipilimumab monotherapy in melanoma brain metastases showed a pooled intracranial objective response rate of 9.0%, while pembrolizumab monotherapy in melanoma brain metastases demonstrated an intracranial objective response rate of 22.0–40.0%. Nivolumab monotherapy in NSCLC brain metastases had a pooled objective response rate of 14% (77).

For GBM, eight studies were included, encompassing phase I and II trials and retrospective analyses. The median survival of GBM patients treated with ICIs across these studies was 7.3 months, with a median progression-free survival of 2.1 months (77). As seen in multiple phase III clinical trials, there was no survival benefit in treating GBM patients with nivolumab. In the clinical trial CheckMate 143, median overall survival in patients treated with nivolumab was not different from those patients treated with bevacizumab (78). In the CheckMate 498 clinical trial, MGMT-methylated GBM patients treated with standard therapy of TMZ and radiotherapy survived longer than those patients treated PD-1 blockade and concomitant radiotherapy (79). Similarly, nivolumab with TMZ and radiotherapy were not superior to radiotherapy, TMZ or placebo in newly diagnosed MGMT-methylated GBM (80).

### Chimeric antigen receptor engineered T cells

CAR T cells are an immunotherapy in which patients’ CD8 T cells are harvested and genetically modified to produce receptors that bind to antigens on the surface of cancer cells. After these CAR-T cells are expanded into millions of copies in the laboratory, they are re-introduced into the patient to recognize and kill cancer cells that carry the target antigen on the cell surface. Since 2017, six CAR T cell therapies have been approved by the FDA to treat lymphomas, leukemia, and multiple myeloma. Because CAR T cell therapy has been so successful in treating blood-borne cancers, neuro-oncologists have been hopeful CAR T cells might treat solid tumors such as GBM.

There are several target molecules expressed on GBM cells including EGFR/EGFRvIII, HER2, B7-H3, and CSPG4 that serve as targets for CAR T cells (81). Our team at City of Hope has designed interleukin 13 receptor alpha-2 (IL13Rα2)-targeting CAR T cells to treat GBM. IL-13 is a cytokine that T helper cells release to regulate inflammation. Binding to biological receptor, IL13Rα1, results in activation of the JAK/STAT pathway. However, when binding to its high-affinity decoy receptor IL13Rα2, a protein that is expressed in over 75% of GBMs and not expressed in normal brain, IL-13Rα2 leads to activation of the rapamycin pathway (82, 83), resulting in increased tumor invasiveness, and therefore poorer prognosis (84). Recent clinical trials utilizing CAR T cells to target CD19 led to extraordinary remission in relapsed or refractory B-cell lymphomas (85, 86), and the FDA has approved this treatment for pediatric (85) and refractory adult (87) acute lymphoblastic leukemia. Because hematological tumor cells can be accessed intravascularly, CAR T cell therapy is easily administered via an intravascular route, and upon delivery, can have direct contact with target tumor cells. In contrast, CAR T therapy for GBM has had marginal efficacy, mostly due to showing antitumor effects in only a subset of patients.

Many hindrances reduce CAR T efficacy for brain tumors. For example, GBM tumors themselves actively contribute to immune suppression through a host of well-orchestrated strategies within a harsh TME. Once CAR T cells reach target tumor cells, the microenvironment may suppress their activity and proliferation by expressing inhibitory cell-surface molecules (such as PD-L1, CD95) (88) or by releasing immunosuppressive tumor-derived soluble factors and cytokines (such as prostaglandin E2, IL6, IL10, and TGFβ) (89). The TME also preferentially promotes the trafficking of suppressive cell populations, such as regulatory T cells (T_regs_), tumor-associated macrophages, microglia, and myeloid-derived suppressor cells (83, 90, 91).

## Tumor treating fields

Tumor Treating Fields (TTFields) therapy is an innovative treatment modality for GBM, that is non-invasive and can be administered alongside standard treatments such as chemotherapy and radiation therapy. TTFields therapy involves the use of low intensity alternating electric fields delivered via transducer sticker arrays placed on the patient’s scalp. TTFields therapy is an antimitotic approach that disrupts the division of glioblastoma cells and their organelle assembly. This is achieved through the application of low intensity alternating electric fields directly to the tumor site. TTFields therapy is its favorable safety profile, as it primarily targets dividing cancer cells while sparing healthy brain tissue from damage.

Clinical studies evaluating the efficacy of TTFields therapy in GBM have shown relatively promising results. The landmark EF-14 was a randomized clinical trial involving 695 patients with GBM who had completed initial radiochemotherapy, were randomized to the addition of TTFields to maintenance temozolamide. The results demonstrated a significant improvement in both progression-free survival (6.7 months in the TTFields plus temozolomide group and 4.0 months in the temozolomide-alone group) and overall survival (median overall survival was 20.9 months in the TTFields-temozolomide group vs 16.0 months in the temozolomide-alone group (HR, 0.63; 95% CI, 0.53–0.76; P < .001)) in newly diagnosed glioblastoma patients who received TTFields therapy in addition to standard chemotherapy compared to those who received chemotherapy alone (92). Additionally, TTFields therapy has been associated with a favorable quality of life profile, with main risk.

While TTFields therapy represents a significant advancement in treating glioblastoma, ongoing research aims to optimize its efficacy and explore its potential in other cancer types. Studies are investigating various aspects of TTFields therapy, including optimal treatment schedules, combination with other therapeutic modalities, and mechanisms of action to enhance its anti-tumor effects further. As TTFields therapy continues to evolve, it holds promise as a valuable addition to the armamentarium of treatments for glioblastoma and potentially other malignancies, offering patients a novel and well-tolerated therapeutic option.

## Immunomodulatory agents

Recently, cytokines such as IFN-α, TNF-α, and IL-12 have been used as novel therapeutic approaches to treat GBM treatment, aiming to alter the immunosuppressive TME to evoke an effective antitumor immune response (93). IFN-α, for instance, has been reported to enhance T cell and macrophage activity and inhibit tumor angiogenesis and immune-suppressive gene expression (94). Conversely, TNF-α fosters dendritic cell maturation and subsequent T cell activation, while IL-12 is associated with improved CAR-T cell functionality, increased infiltration of CD4+ T cells, and reduced T-regulatory cell frequency within the tumor microenvironment (95, 96).

However, IFN-α therapy is hindered by its significant systemic toxicity and limited efficacy at maximum tolerated doses, as evidenced by clinical trials reporting adverse effects such as hyperthermia, shivering, headaches, gastrointestinal symptoms, and orthostatic hypotension (97). This restricts its current use, highlighting the need for future strategies that combine IFN-α with other treatments to enhance both efficacy and tolerability (93).

IL-6 is a multifaceted cytokine that orchestrates several biological processes, such as the acute phase response, immune defense against infections, leukocyte infiltration at inflammation sites, leukocyte maturation, and endothelial cell characteristics (98). The IL-6 signaling pathway initiates with IL-6 binding to membrane-bound IL-6R (gp80) present on various cells like hepatocytes, neutrophils, monocytes, B cells, and T cells. Subsequently, the IL-6-IL-6R complex binds to membrane-bound gp130, expressed ubiquitously by all cells, forming an activated IL-6 receptor that triggers intracellular signaling. This classic IL-6 signaling pathway leads to anti-inflammatory responses. Alternatively, IL-6 can also act via the trans-signaling pathway, binding to soluble forms of IL-6R, and then binding to membrane-bound gp130, activating target cells lacking membrane-bound IL-6R and inducing pro-inflammatory responses (99, 100). Both pathways activate Janus kinases (JAKs) and signal transducer and activator of transcription (STATs) to modulate cellular responses (98).

Overexpression of IL-6 has been linked to poor survival in GBM patients based on data from The Cancer Genome Atlas (TCGA) and the Repository of Molecular Brain Neoplasia Data (REMBRANDT) (101). Within the GBM TME, IL-6 is secreted by tumor cells, glioma-associated microglia/macrophages (GAMs), and tumor-associated endothelial cells (102, 103), with its expression induced by treatments such as chemotherapy (104) and radiotherapy (105). IL-6 exerts its effects through IL-6R expressed on tumor cells and GAMs, promoting immunosuppressive GAMs and impairing T cell functions. Treatment with anti-IL-6 siltuximab, anti-IL-6R tocilizumab, or STAT3 inhibitor Stattic has been shown to mitigate PD-L1 expression and T cell apoptosis, underscoring the role of IL-6 signaling in regulating T cell responses via GAMs (28). Additionally, IL-6 sustains tumor progression by directly influencing glioblastoma cells to activate anti-apoptotic pathways, induce autophagy, and promote invasion through mechanisms involving MMPs and fascin-1 (106).

## Viral immunotherapy

Oncolytic viruses (OVs) are weakly pathogenic viruses designed to selectively infect, replicate within, and kill tumor cells through apoptosis while avoiding infection of healthy cells (107). OVs stimulate the innate immune system through pathogen-associated molecular patterns and pattern recognition receptors that recruit immune cells such as neutrophils, macrophages, natural killer cells, Th1 cells, leading to cell lysis (108). This response also leads to an adaptive immune reaction to new cancer antigens, therefore possibly propagating long-term anti-tumor immunity (109). Moreover, OVs are able to convert the GBM TME from “cold” to “hot” by triggering inflammation, and improve the outcome of immune checkpoint inhibitors (110). OVs utilizing modified herpes simplex virus, adenovirus, measles virus, parvovirus, reovirus, and poliovirus, amongst others, are being studied in clinical trials against GBM worldwide (93). Because OVs are noted to have oncolytic activity but low specificity, various methods have been employed to improve tumor-specificity such as deleing virulence genes, adding tumor-specific promotors, or adding tumor suppressor gene miRNAs (111).

More than 20 oncolytic viruses have been evaluated in clinical trials for GBM. Key examples include HSV-1, adenovirus, reovirus, measles virus, Newcastle disease virus, and poliovirus (112). To circumvent the limitations imposed by the BBB, innovative delivery methods such as convection-enhanced delivery (CED) have been utilized. CED is particularly used for the recombinant nonpathogenic polio-rhinovirus chimera (PVSRIPO) and works by using a pressure gradient within a catheter to deliver therapeutic agents into the interstitial spaces of the brain parenchyma (113). Achieving efficient and safe delivery of oncolytic viruses is critical for successful virotherapy. The difficulty of delivering these viruses to the CNS and their potential elimination by the immune system has made intratumoral delivery the preferred approach. However, for optimal outcomes, the oncolytic virus should ideally be administered systemically to reach both primary and metastatic tumor sites, especially in the cases of multifocal GBM.

The predominant immune cells in the GBM TME are macrophages originating from peripheral monocytes, known as tumor-associated macrophages (TAMs) (114). TAMs primarily express the M2 surface marker and produce IL-10 and TGF-β with STAT3 expression, promoting an immunosuppressive “cold” TME (115). Additionally, T cell exhaustion, characterized by the upregulation of inhibitory molecules and increased Treg cells, is a hallmark of GBM (116). Moreover, expression levels of immunomodulatory chemokines CXCL2, CX3CL1, CCL5, and CCL2 are elevated in GBMs (117). Consequently, OVs could enhance alternative therapies by reversing these conditions to promote greater immune infiltration and improved tumor destruction (118). For example, delivering concomitant OVs with ICIs has shown promising outcomes. Infection with the measles virus in GBM models resulted in increased PD-L1 expression (119); and using both measles as well as anti-PD-1 antibodies resulted in longer survival in murine glioma models compared to survivals in animals treated with each therapy alone (120). Similarly, OVs can improve CAR T cell therapy against solid tumors (112). Care must be taken however, as application of a vesicular stomatitis virus encoding IFNβ resulted in the reverse outcome on CAR T cells targeting EGFRvIII in B16 murine glioma tumors (121). Further understanding of the complex interplay between OVs and other immunological treatments must be further characterized prior to use in larger-scale clinical trials.

## Utilizing intratumoral bacteria

With ever-advancing technologies to study the TME, intratumoral bacterial and fungal biomasses have been characterized in multiple tumor types (122, 123). Intratumoral microbiota generates anti-tumorigenic effects by increasing antigen presentation, activating NK and T cells, improving immunosurveillance, and ultimately generating metabolites that suppress tumor progression (124). Intratumoral bacteria can induce potent immunogenic antitumor effects and significantly extend survival rates with effective immunological memory in various murine cancer models (125), such as against melanoma (126). Memory T cells pre-stimulated by bacteria-derived or tumor-derived antigens within GBM are found in peripheral blood and tumors (127). There is cross-reactivity between human tumor antigens and bacterial antigens due to molecular mimicry, allowing tumor antigen recognition by CD4+ T cells (127) and CD8+ cytotoxic T cells (128). Indeed, GBM contains distinct microbiota compared to adjacent normal tissues (129). Bacteria-specific peptides obtained from normal cell lines or GBM tumor samples are presented via MHC II molecules. After stimulation by these bacterial peptides, tumor-infiltrating lymphocytes secrete more pro-inflammatory cytokines and recruit CD8+ T cells to enhance tumor-killing (130). For patients where intratumoral bacterial nucleic acids or peptides are detected in very low quantities, subcutaneous injection of tumor antigen-engineered commensal bacteria to elicit distant anti-tumor immune responses is a novel and safe approach (126), and is an exciting option for GBM treatment (131). Through the subcutaneous delivery of lysed-engineered bacterial peptides, epidermal Langerhans cells further present tumor peptides in MHC molecules, allowing distant primed CD8+ T cells to enter the circulation and infiltrate tumors. This vaccination approach avoids HLA restriction (132) and is an exciting option to be used alone or in combination with other tumor vaccine strategies (131).

## Strategies to improve immunotherapies

Various approaches have been suggested to address hypoxia in cancer, including inhibition of HIF signaling, the use of hypoxia-activated prodrugs (HAPs), targeting downstream pathways like the unfolded protein response (UPR) and mTOR, as well as metabolic interventions (133). Considering hypoxia’s central role in regulating tumor progression and immune suppression, it is plausible to explore hypoxia as a potential target in combined cancer immunotherapy. This section delves into the manipulation of hypoxia to enhance the effectiveness of cancer immunotherapy, drawing insights from both pre-clinical and clinical studies. Please refer to **Box 1** for more details.

### Hypoxia-activated prodrugs

HAPs, also referred to as bioreductive prodrugs, are inert compounds converted into active substances by enzymatic reduction, selectively targeting hypoxic TMEs (134). Despite promising pre-clinical hypoxic cytotoxicity, some HAPs demonstrated disappointing clinical efficacy, leading to their discontinuation. Tirapazamine (TRP) is the first HAP that showed clinical safety in 1994 (135), however, despite optimistic preclinical findings, phase III studies showed no therapeutic benefit compared to chemotherapy alone or chemoradiotherapy (136). Additionally, the mitomycin C derivative prodrug praziquantel (EO9) displayed efficacy in superficial bladder cancer patients (137), however, a phase III clinical trial evaluating the efficacy and safety of EO9 (NCT01469221) was unfortunately discontinued (134). A second generation TRP analogue, SN30000, demonstrated antitumor effects in xenograft models (138), and the New Zealand group working on this analogue hope to generate Phase I clinical data from this.

Notably, evofosfamide (TH-302), a TH-302, a prodrug of the cytotoxic alkylating agent bromo-isophosphoramide mustard, is favorably activated in hypoxic conditions. TH-302 has shown non-lymphotoxic properties and compatibility with immunotherapy (139), specifically with PD-1 and CTLA-4 blockade. Combining TH-302 with these immunotherapies achieved remarkable tumor cures and prolonged survival in prostate cancer mouse models (140). A recent phase I clinical trial (NCT03098160) evaluated TH-302 in combination with ipilimumab against various cancers, including melanoma, pancreatic, and prostate tumors, which showed no new safety issues and showed evidence of therapeutic activity (141). Encouraging results from a Phase II clinical trial involving TH-302 with doxorubicin for soft tissue sarcoma (142) led to an international, open-label phase III study using TH-302 in combination with doxorubicin as first-line therapy for locally advanced soft-tissue sarcoma (NCT01440088). Unfortunately, the combination did not show any overall survival benefit compared to those patients treated with single-drug doxorubicin alone (143). More recently, a Phase II clinical trial using TH302 for recurrent bevacizumab-refractory GBM showed that progression-free survival at 4 months following TH302-bevacizumab treatment was 31%, significantly higher than the historical rate of 3% (144).

### Drugs targeting HIF signaling pathways

Drugs targeting hypoxia-inducible factors (HIFs) are under development, and are classified by their interference with HIF dimerization, DNA binding, mRNA or protein expression, or degradation (145). Combining classical chemotherapeutic agents, such as cisplatin, doxorubicin, and cyclophosphamide, with HIF pathway inhibition shows promise in enhancing antitumor immune responses (146). Studies indicate that downregulating HIF-1 expression by antisense HIF1-α enhances NK cell and CD8 T cell-mediated antitumor immunity, resulting in anti-tumor activity (147). PX-478 (S-2-amino-3-[4 V-*N*,*N*,-bis(2-chloroethyl) amino]-phenyl propionic acid N-oxide dihydrochloride) is a small-molecule that suppresses HIF-1α translation that, when given with a dendritic cell-based vaccine, dramatically reduces breast cancer in mice (148). Similarly, combining HIF-1-mediated ectoenzyme ENTPD2 inhibitors with anti-CTLA-4/PD-1 ICIs significantly increased T-cell infiltration in hepatocellular carcinoma tumors and extended survival in tumor-bearing mice (149).

Additional small molecules, including the Hsp90 inhibitor (17-AAG) (150), digoxin (151), and 2-methoxy estradiol (2ME2) (152), act non-specifically on HIF by inhibiting its synthesis or stabilization and have shown tumor inhibition. In contrast, PT2385 and its derivative, PT2399, uniquely bind to HIF2, disrupting its heterodimerization with HIF-β (153). The efficacy of PT2399 was validated in a preclinical model of clear-cell renal cancer cells with heightened HIF activation due to VHL mutation (154). Results from a recent Phase I clinical trial demonstrated the safety and efficacy of PT2385 as a direct HIF-2 inhibitor in locally advanced or metastatic clear cell renal carcinoma (155). More recently, a single-arm open-label phase II study of patients with recurrent GBM was treated with oral PT2385, which had limited anti-tumor effect, in part because of variable drug exposure (156). PT2977 (MK-6482), a more potent second-generation HIF-2 inhibitor, is currently undergoing a Phase III clinical trial in advanced renal cell carcinoma (NCT04195750) (157). It’s crucial to note that none of these studies evaluated tumor oxygenation status, emphasizing the need for further investigation to determine whether this treatment is more advantageous for specific patient populations exhibiting high HIF-2 expression or elevated tumor hypoxia.

### Metabolic regulation

Several metabolic targets are being studied to reduce tumor hypoxia and improve treatment sensitivities to radiation as well as chemotherapies and immunotherapies. Oxygen is utilized by mitochondria through oxidative phosphorylation and the electron transport chain. Reducing cellular oxygen consumption rates by inhibiting oxidative phosphorylation increases oxygen in the TME and improves oxygen diffusion into hypoxic areas (157). One strategy to reduce tumor hypoxia in tumors is to lower the oxygen consumption rate in tissue. Reducing oxygen consumption by just 30% in poorly perfused tumor regions was more effective at reducing tumor hypoxia than increasing blood flow or increasing oxygen levels in the blood (158). Reducing oxygen consumption is an intriguing strategy because it also circumvents the need for drugs to diffuse into poorly oxygenated areas and can be used across multiple tumor types (157).

Metformin is an anti-diabetic biguanide that became an interesting metabolic targeting drug when it was noted that diabetic patients on metformin had a lower cancer incidence than diabetics on other medications, or non-diabetics (159). Metformin inhibits mitochondrial complex 1, which activates AMPK and inhibits mTOR, which overall reduces oxygen consumption rate (160). Although preclinical murine studies showed that metformin improved tumor oxygenation and increased radiotherapy sensitivity (161), metformin was not shown to benefit patients with non-small cell lung cancer in clinical trials (162, 163). There have been a number of clinical studies performed that had shown promising results in treating tumors, however due to insufficient sample sizes, the results have not been conclusive [for review see (164)].

Carbonic anhydrase isoform IX (CAIX), a cell-surface pH regulatory enzyme upregulated by HIF-1α and HIF-2α, is another exciting metabolic target. CAIX activates glycolysis, contributing to immune suppression in various solid malignancies (165). Targeting CAIX with monoclonal antibodies or small-molecule inhibitors, such as SLC-0111, decreases glycolytic metabolism and ultimately leads to increased immune activity (166). Since 1998, various clinical trials have been used to study anti-CAIX monoclonal antibodies against renal cell cancer, with various results such as complete responses, maintaining stable disease, and partial responses (see (167) for review). Blocking CAIX renders tumors more sensitive to ICIs due to enhanced Th1 responses and reduced tumor invasiveness. Therefore, inhibition of CAIX, in combination with immunotherapy, proves to be a potential strategy for improving clinical outcomes in hypoxic tumors (168).

Hypoxia-induced accumulation of extracellular adenosine leads to an immunosuppressive TME. Adenosine-A2A receptors (A2AR) is a G-protein-coupled receptor expressed in many immune cells such as cytotoxic T cells, macrophages, and regulatory T cells (169). In a pre-clinical study of squamous cell carcinoma, application of A2AR-blocker, SCH5826, decreased CD4+ T regs and promoted a CD8+ T cell-mediated response that reduced tumor growth (170). Indeed, inhibiting A2AR resulted in improved efficacy of chemotherapy and immunotherapy in multiple cancers such as lung adenocarcinoma, renal cell cancer, paraganglioma, and pheochromocytoma (171–174).

Given that metabolic reprogramming is so crucial to cancer cell growth and infiltration of immunosuppressive myeloid cells into the tumors, Khan et al. screened metabolic small-molecule compounds for impaired macrophage migration and determined that lactate dehydrogenase (LDH) inhibitor stiripentol, an anti-seizure medication was a top hit. They further characterized that LDHA-dependent ERK pathway leads to activation of YAP1/STAT3 and upregulation of chemokines CCL2 and CCL7. The team further determined that both genetic and pharmacological blockade of LDHA-mediated tumor-macrophage symbiosis both suppressed macrophage infiltration in murine GBMs as well as reduced tumor progression. LDHA-mediated glycolysis also results in chemo-radiotherapy resistance, therefore, targeting LDHA and its downstream pathways is a further attractive strategy to combat GBM (175, 176)

### Supplemental oxygenation

Supplemental oxygen therapy, combined with existing immunotherapies, has the potential to decrease tumor hypoxia and extracellular adenosine accumulation. Respiratory hyperoxia with 60% oxygen enhances intra-tumoral infiltration of CTLs and improves antitumor responses when given with dual PD-1 and CTLA-4 therapy in mouse models (177). Indeed, adoptive immunotherapy combined with respiratory hyperoxia led to the complete obliteration of fibrosarcoma tumors in mice (177). The approach of using supplemental oxygen as an immunological co-adjuvant warrants further investigation in combination with existing cancer immunotherapies. Conversely, inhibiting both oxygen consumption and tumor hypoxia with metformin is correlated with improved efficacy of PD-1 blockade immunotherapy (178).

Hyperbaric oxygen (HBO) treatment delivers 100% oxygen gas at 2–4 times normal atmospheric pressure, which increases a patient’s saturated hemoglobin. In the 1970s and 80s, clinical trials showed that HBO treatment delivered before radiation treatment improved local control of squamous cell carcinoma and cervical cancer. However, HBO treatment had technical limitations related to decompression of the chamber, which led to tissue damage and therefore has since fell out of favor (179). Carbogen gas (composed of 95% oxygen and 5% carbon dioxide) breathing became an alternative to HBO as it could promote oxygen delivery via vasodilation and increased blood flow (180), but does not require an oxygen chamber, which allows the gas to be more easily delivered. Carbogen gas breathing showed inconsistent results, however, which might be related to the duration of time subjects breathed the gas prior to treatments (181).

### Vessel normalization

Hypoxia, resulting from abnormal tumor vascularization, upregulates VEGF through HIF-1, creating a detrimental cycle of worsening hypoxia (139). VEGF/VEGFR-targeted therapies induce anti-angiogenic effects, reducing hypoxia and supporting the immune response. However, monotherapy with angiogenesis inhibitors can exacerbate tumor hypoxia, rendering both chemotherapies and radiation treatment less effective (182). Vascular normalization, achieved with low-dose anti-angiogenic agents, enhances immunotherapy efficacy and reduces toxicity. Clinical trials combining PD-1 blockade with anti-angiogenic agents, such as bevacizumab (183) or Lenvatinib (184), show promise in various cancers, including melanoma and hepatocellular carcinoma. In fact, ICIs induce interferon-γ production from CD4+/CD8+ T cells, play an important role in the normalization of blood vessels, and work as an anti-angiogenesis therapy themselves (185). Based on Phase III clinical trial results, the FDA has approved combinations of PD1/PD-L1 antibodies with anti-VEGF/VEGFR agents in lung and renal cancers (186). Additionally, there is much promise in utilizing anti-angiogenesis therapy with immunotherapy to augment radiotherapy (185). Nonetheless, the impact of hypoxic stress on tumor heterogeneity remains a question, emphasizing the need for tailored approaches based on tumor type, patient status, and reliable biomarkers.

In summary, the combination of hypoxia-targeting strategies with immunotherapy holds promise for enhancing therapeutic outcomes in cancer patients. However, the diverse responses to hypoxia-modifying therapy highlight the importance of personalized medicine, necessitating the identification of specific hypoxia markers for targeted therapies and robust anti-tumor immune responses.

**Box 1: Advantages and disadvantages of methods to improve immunotherapies.**

Inhibition of HIF signaling

Advantages: Combining classical chemotherapeutic agents, such as cisplatin, doxorubicin, and cyclophosphamide, with HIF pathway inhibition shows promise in enhancing antitumor immune responses.

Disadvantages: studies on the efficacy of PT2399, PT2385 and PT2977 HIF-2 inhibitors have not evaluated tumor oxygenation status, emphasizing the need for further investigation to determine whether this treatment is more advantageous for specific patient populations exhibiting high HIF-2 expression or elevated tumor hypoxia.

Hypoxia-activating prodrugs (HAPs)

Advantages: HAPs, also referred to as bioreductive prodrugs, are inert compounds converted into active substances by enzymatic reduction, selectively targeting hypoxic TMES Disadvantages: Despite promising pre-clinical hypoxic cytotoxicity, some HAPs demonstrated disappointing clinical efficacy, leading to their discontinuation.

Manipulation of downstream pathways:

Advantages: Supplemental oxygen therapy, combined with existing immunotherapies, has the potential to decrease tumor hypoxia and extracellular adenosine accumulation. Vascular normalization, achieved with low-dose anti-angiogenic agents, enhances immunotherapy efficacy and reduces toxicity and the FDA has approved combinations of PD1/PD-LI antibodies with anti-VEGF/VEGFR agents in lung and renal cancers.

Disadvantages: Carbogen gas, a supplemental oxygen therapy, can promote oxygen delivery, but has shown inconsistent results which might be related to the duration of time subjects breathed the gas prior to treatments. For vessel normalization, while there is much promise in utilizing anti-angiogenesis therapy with immunotherapy to augment radiotherapy, the impact of hypoxic stress on tumor heterogeneity remains a question, emphasizing the need for tailored approaches based on tumor type, patient status, and reliable biomarkers.

Interventions leading to metabolic changes:

Advantages: Reducing cellular oxygen consumption rates by inhibiting oxidative phosphorylation increases oxygen in the TME and improves oxygen diffusion into hypoxic areas.

Disadvantages: More clinical studies are needed to test efficacy of Metabolic targeting drugs, such as Metformin, inhibition of metabolic targets such as CAIX and A2AR, and metabolic small- molecule compounds such as LDH.

## Future directions

Future advances leveraging RNA-seq and single-cell RNA sequencing techniques, along with bioinformatics and AI technology, hold immense potential in unraveling the intricacies of GBM TME. RNA-seq provides a high-throughput method to analyze gene expression profiles, allowing researchers to decipher the molecular patterns associated with different cell types within the TME. Moreover, sc-RNA-seq enables the dissection of individual cells, providing unprecedented insights into the heterogeneity and cellular interactions driving GBM progression and therapy resistance (53).

By harnessing the power of bioinformatics and AI, researchers can sift through vast amounts of genomic data generated from RNA-seq and sc-RNA-seq experiments to identify key molecular players and signaling pathways implicated in GBM pathogenesis. Advanced computational algorithms can integrate multi-omics data, such as genomics, transcriptomics, proteomics and epigenomics, to construct comprehensive models of the GBM TME. These models not only enhance our understanding of tumor biology but also facilitate the identification of novel therapeutic targets and the development of personalized treatment strategies. Furthermore, AI-driven approaches enable predicting patient outcomes and therapeutic responses, paving the way for precision medicine in GBM management.

## Conclusion

It’s important to understand the dynamics of hypoxia in the GBM TME to develop targeted therapies that address the complexities of such an environment. The unique features of the TME, such as the presence of CSCs, interactions with stromal and immune cells, and the vascular and hypoxic niches, can be targeted to develop more effective and personalized treatments. However, developing clinically viable therapies requires overcoming the challenges posed by the remarkable heterogeneity inherent to GBM. Advances in precision medicine, immunotherapy, and innovative drug delivery systems are being explored to design therapies that account for the diverse elements of the GBM TME and to improve patient outcomes.

## Author contributions

LF: Writing – original draft, Writing – review & editing.
